# Microstructure Evolution of Super304H Steel Used in a Service Power Station Boiler

**DOI:** 10.3390/ma17225518

**Published:** 2024-11-12

**Authors:** Xiaoxin Wang, Baohe Yuan, Jianbin Li, Guoxi Chen

**Affiliations:** 1 Henan Boiler and Pressure Vessel Inspection Technology Research Institute, Zhengzhou 450045, China; wangxx0330@163.com (X.W.);; 2 North China University of Water Resources and Electric Power, Zhengzhou 450045, China

**Keywords:** Super304H, burst pipe, surface morphology, Vickers hardness

## Abstract

The microstructure and structure of a Super304H superheater steel pipe after 47,000 h were analyzed by metallographic microscope, scanning electron microscope (SEM), and EDS, and its mechanical properties were measured by hardness meter. The results show that the austenitic grains appear on the outer wall of Super304H steel pipe after service, while the SEM and metallographic microscope tests show that the outer wall particles are coarse. There is an obvious corrosion layer on the outer surface, and the thickness of the corrosion layer on the windward surface is significantly higher than that on the leeward surface. The inner surface is refined and the hardness of the material is significantly increased; the outer surface, the inner surface, and the center all grow abnormally. In this case, the room temperature tensile strength and impact performance of the rough crystal area of the outer wall of the Super304H steel pipe are reduced and fracture along the crystal. Supervision should be strengthened to eliminate the safety risks caused by the abnormal growth of the outer wall austenite grain. The results of crystal phase microscopy show that the main structure of the material still maintains the basic structure of austenitic steel, and particle aggregation mainly occurs in the sub-inner layer of the inner and outer surface. Compared with the lee surface, the middle body structure is basically the same, but whether the thickness of the corrosion layer on the inner surface or the outer surface increases, the deformation degree of the deformation layer is greater. The hardness measurement finds that the hardness of the corrosion layer is caused by the increase in Super304H steel surface stress. In case of pipe explosion accident, the highest chance of pipe explosion here should be closely observed.

## 1. Introduction

In recent years, clean power generation methods such as wind power generation and solar power generation have developed rapidly, but thermal power generation still occupies a dominant position in the national power generation system. According to statistics, by the end of 2023, thermal power installed capacity accounted for 48% of China’s power generation structure, and the annual power generation capacity was 6231.8 billion kWh, up 6.1% year on year, accounting for 69.95% of China’s total power generation in that year, ranking first. With the development of power generation technology, ultra-supercritical units have been applied to power generation, which greatly improves the efficiency of power generation and reduces the consumption of fossil energy and greenhouse gas emissions [1,2,3,4,5]. However, the working environment of the ultra-supercritical unit is harsh, and the steel pipes such as the reheater and superheater operate under high temperatures above 600 °C for a long time, so higher requirements are put forward for heat-resistant steel pipes. As a new type of fine crystal austenitic heat-resistant steel, Super304H is widely used in high-temperature superheater and reheater steel pipe of ultra-supercritical units with good structure stability, high-temperature strength, and anti-steam oxidation performance [6,7,8]. Austenitic heat-resistant steel Super304H is a kind of austenitic steel that increases the content of molybdenum, chromium, and nickel on the basis of TP304 heat-resistant steel, adding about 3 wt% Cu and a small amount of Nb. The addition of Cu, Cr, and Ni content allows it to have good high-temperature corrosion resistance and steam corrosion resistance, and also have good welding performance [9,10].

The structure aging of heat-resistant steel Super304H is mainly manifested by the coarsening of the second phase, and the growth of austenite grains will precipitate from Super304H at high temperature, which involves a rich Cu phase, MX phase (Nb (C, N)), Z phase (NbCrN), and M_23_C_6_ [7,8,11,12]. These precipitation phases allow it to have good high temperature strength. Cu precipitation is the main contribution to improve the material strength. The fine rich Cu precipitated phase has a bcc structure colocalized with the matrix. Then the relationship of the precipitated phase and the matrix changes from colattice to fcc structure with Kurdjumov-Sachs (K-S) orientation [13,14]. However, before the loss of the colattice interface between the Cu phase and the matrix, the dislocation of the Cu phase particles increases the substrate hardness [15]. In Super304H steel with long time aging at 700 °C, it was found that M_23_C_6_ gathered and grew with time and gradually continuously distributed, while the MX (mainly Nb (C, N)) phase distributed all the time. Under extreme conditions, the abnormal growth of austenite grains (crude crystal) occurs in advance, but the precipitation of the second phase is not obvious. The precipitation behavior of the second phase particles in the service rough crystal Super304H steel sample has a great influence on the mechanical properties of steel pipes.

In this paper, according to the service environment of heater materials, the persistence performance and organization evolution of Super304H steel are studied under the joint action of flue gas/coal ash-stress under the temperature of more than 600 °C. The boiler operation time is 47,000 h, the maximum continuous evaporation is 2044 t/h, reheat steam flow, 1723 t/h, the superheater outlet working pressure is 26.15 MPa, the superheater outlet steam temperature is 605 °C, the reheater outlet working pressure is 5.96 MPa, and the reheater outlet steam temperature is 603 °C.

## 2. Materials and Methods

The supplied Super304H steel tubes were taken from a superheater spray pill. The outer diameter of tube is 48 mm and the wall thickness is 7 mm. A steel tube was cut and measured on windward and leeward surfaces and base. The composition of the steel tube is listed in Table 1.

The test samples were polished to the mirror and dried after ethanol solution. Using copper sulfate-hydrochloride solution (CuSO_4_: HCl: H_2_O: 5 g: 20 mL: 20 mL) for 10–30 s, we placed the sample under the optical microscope (OM, Olympus BX51, Olympus (China) Co., Ltd., Shanghai Branch, Shanghai, China) to observe the microscopic structure of the specimen. Microstructure and grain boundary were characterized by a gold microscope. Using MH-3 micro Vioner hardness meter, we measured in service state Super304H the hardness of the inner and outer walls of the heat-resistant steel tube. The morphology and microstructure of the samples were characterized by scanning electron microscopy (SEM), and the elemental composition and elemental distribution of the samples were analyzed using energy dispersive spectroscopy (EDS).

## 3. Results and Discussion

**Figure 1:**
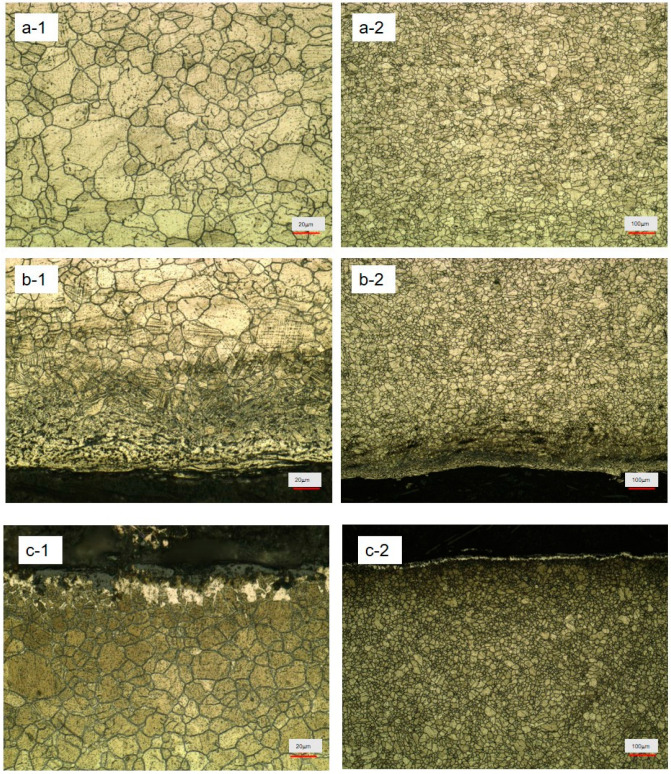
Pretreated service Super304H leeward surface gold phase microscope: (**a-1**,**a-2**) center, (**b-1**,**b-2**) medial, (**c-1**,**c-2**) outside.

**Figure 2:**
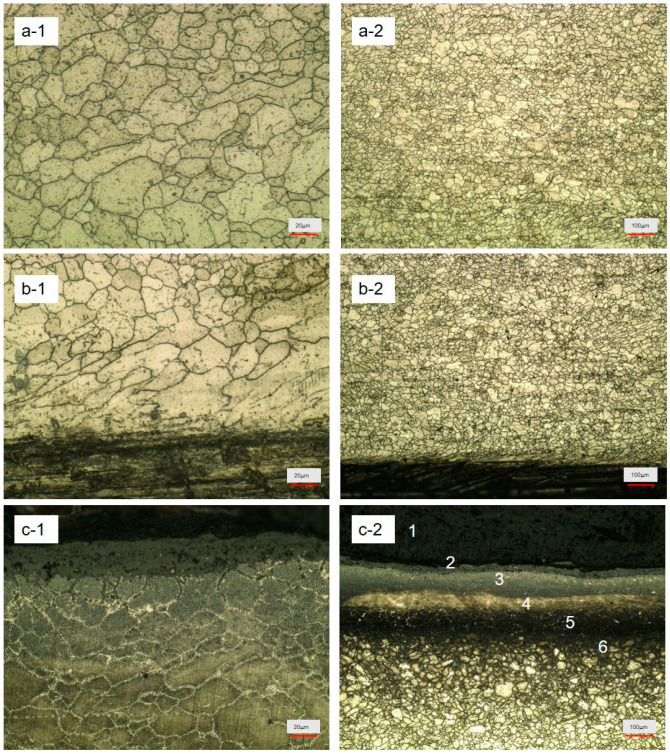
Pretreated service Super304H windward gold microscope: (**a-1**,**a-2**) center, (**b-1**,**b-2**) medial, (**c-1**,**c-2**) outside.

Figure 1 presents a picture of the gold phase microscope of the Super304H steel pipe after pretreatment. Figure 1(a-1,a-2) shows the lee plane pipe middle part of the gold phase microscope images. The center austenite grain size was uniform between 10–35 μm, or 50–100 μm larger grain, with clear boundaries, but some small particles fused into the large grain trend. This shows that there is larger grain size performance decline with the increase of service time. Figure 1(b-1,b-2) shows a picture of a gold phase microscope on the inner wall of the Super304H lee plane tube, from the picture. There is a distinct layer of corrosion on the surface of the inner wall. The thickness of the corrosion layer is between 100 and 200 μm. The interface between the corrosion layer and the inner layer is clearly evenly distributed from the corrosion layer to the inner grain. But on the surface, the structure forms a porous link layer. Structurally, the stress is very large to form a protective layer. Between the protective layer and the inner layer grain, there is about 10 μm of high-stress layer. The grains in this layer have obvious stress deformation. A parallel crystalline phase structure is formed along the layers under the lateral stress. There is a protective layer. The transition layer structure is stabilized under the dual protection of high hardness and large stress. After these two layers, it enters the body structure. It can be seen that the inner layer of double protection forms a protective film to make the material more durable.

Figure 1(c-1,c-2) presents the lee plane tube wall part of the gold microscope pictures. Here, the steel pipe outer wall back fire side outside has a coarse grain and wider distribution. There is a fine grain to coarse grain fusion trend, with a coarse austenite grain ‘island’ swallowing tiny austenite grain. From the outside to the inside, the whole field of vision no longer shows the complete structure. From Figure 1(c-1,c-2), it is visible at 500–800 μm that all the steel pipe wall structure formed a wide range of uncontrollable structure, which would require a safety evaluation if for continued use. The surface corrosion layer completely becomes a different structure, and there is no austenite or any steel structure. This structure will lead to pipe aging, which may seriously burst the tube and needs to be closely monitored. According to this direction, burst tube probability will be greatly increased.

Figure 2 presents a mirror picture of the windward microscope of the Super304H steel tube. Figure 2(a-1,a-2) is the middle part of the wind microscope images. Here, the center austenite grain size uniformity is obviously not enough. There are 40–80 μm larger grains throughout the whole field of vision, where small particles fuse into large particles. Compared with the wind, grain fusion is more serious; in the center of the pipe, the wall grain polymerization phenomenon by temperature and stress effect is more obvious. Figure 2(b-1,b-2) shows a picture of the inner wall of the tube of the Super304H windward side. The corrosion layer thickness of the surface of pipe inner wall is more than 150 μm. The interface between the corrosion layer and the inner layer is clear, and the higher stress layer has a greater thickness. The high stress layer forms a layer parallel to the surface of the material. In the inward observation, we found 20–80 μm crystal boundaries within the m thickness range. It is found that the distribution of grains tends to be completely parallel under stress. Through the site investigation, we found that the parallel direction is the direction of water vapor flow. Reviewing the plant operation data shows that the temperature gradient is much higher than the lee plane. We continued to find the grain boundary inside the middle of the tube but the grain size is much larger than the inside. Almost no small grains are found in the entire field of view, and there is significant fusion along one direction between large grains. This influence thickness range is not less than 300 μm.

Figure 2(c-1,c-2) shows a picture of the outer wall of the pipe on the windward side, and the outer wall of the steel pipe shows an obvious layered structure. No less than six layers of structure are formed from outside to inside, and the first layer presents an obvious loose structure, which is a completely corrosive layer, which will fall off under a very weak external force. The second layer is relatively loose on the pipe. There is a clear interval between this layer and the first layer. It is filled with holes but is relatively closely bound with the internal structure, but there are obvious holes at the junction. The third layer shows an obvious grain shape, but the loose structure between grains is not more excessive than small grains. It can be seen from Figure 2c-1 that the link between grains from the inside out is gradually reduced, and the boundary between grains is clear. These no longer have fusion conditions but tend to peel each other; the more on the outside, the more obvious. The grain size of the fourth layer is concentrated at 50–120 μm. There is no obvious gap between the grains, but there are many subtle precipitation phases between the crystal boundary. The crystal boundary is clear, with no obvious fusion trace, and the outer grain size is larger. The fifth layer grain size compared to the fourth layer has a small particle size between much larger ones, located relatively closely. The grain boundary density is much higher than in the fourth layer, but the grain size is much larger than in the sixth layer. Thus, the structure of its mechanical strength is still very poor, or in the scope of complete corrosion layer. In the sixth layer, the grain distribution is uniform in size at 20–40 μm. A small number of particles gather, and the dispersion between particles is significantly higher than the central part (Figure 2(a-1,a-2)). The layer thickness is 400–700 μm, and although the material has a certain mechanical strength, regarding the structure tightness, there are no signs of fusion between particles. With this, we can infer that the hardness will increase, toughness will decrease, and deformation ability is reduced if the load change, temperature, heat impact, and mechanical impact under the double action probability is greatly increased. On the whole, the main structure of the material also maintains the basic structure of austenitic steel, and particle aggregation mainly occurs in the inner layer of the inner and outer surface. Compared with the lee surface, the middle body structure is basically the same, but the thickness of the corrosion layer on the inner surface and outer surface increases, and the deformation degree of the deformation layer is greater. In the case of pipe explosion accident, the highest chance of pipe explosion here should be closely observed.

**Figure 3:**
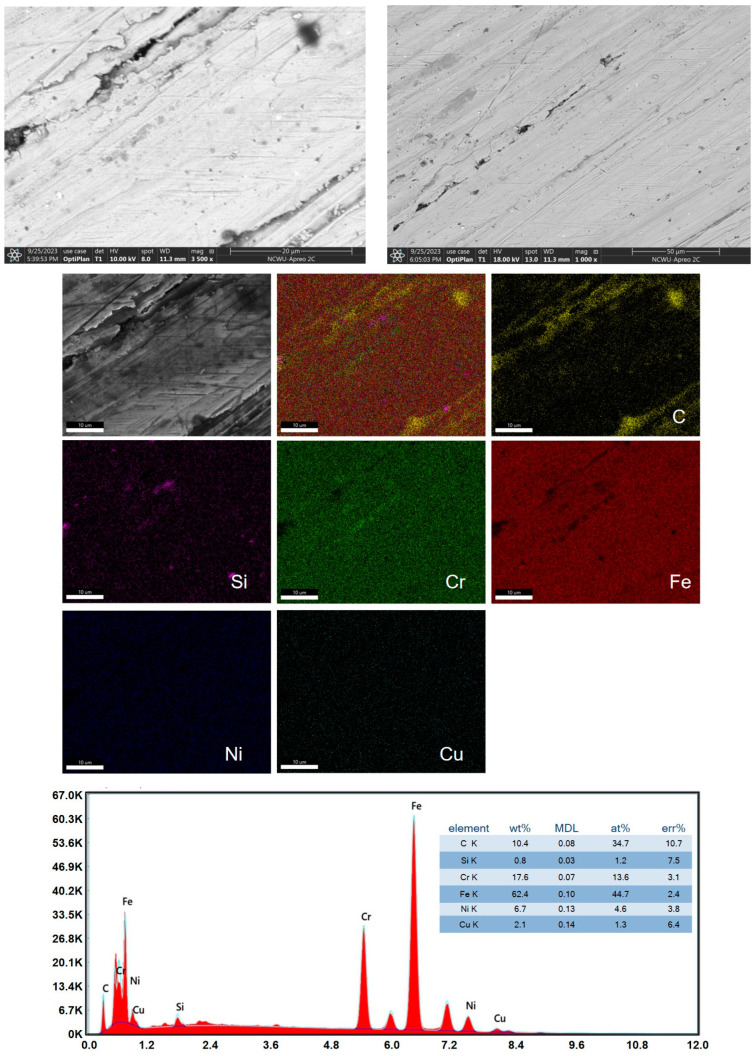
SEM picture and EDS at the central break point.

To further analyze the microscopic structural changes and particle composition of the service Super304H steel tube, we measured the scanning electron microscope pictures and EDS surface scanning at the center cut of Super304H steel tube after 47,000 h of service. Figure 3 presents the picture of SEM and EDS. As can be seen from Figure 3 SEM images, the central steel structure is tight with no obvious defects. From the scratch point of view, the hardness and toughness of the center are basically the same. This indicates that there is no deformation and performance decline in the central site. EDS analysis shows that the main components of Super304H in the steel pipe are C, Si, Cr, Fe, Ni, and Cu, which are consistent with the main components of Super304H steel. From the perspective of surface scanning distribution, Cr, Fe, Ni, and Cu are evenly distributed, but C and Si have an aggregation phenomenon, which indicates that the material has precipitated carbon grains in the process of thermal aging, which is consistent with the literature report that M_23_C_6_ accumulates with the extension of thermal impact time [16].

**Figure 4:**
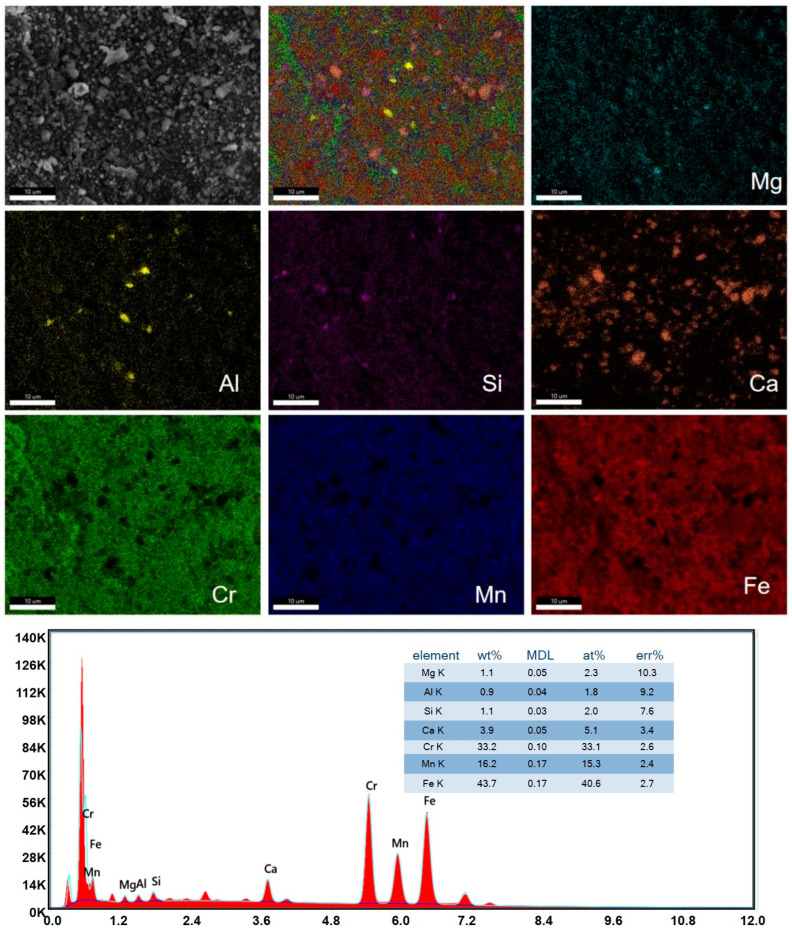
Inner layer surface SEM and EDS.

We measured scanning electron microscopy and EDS surface scans of the inner surface of the Super304H steel tube after 47,000 h of service. As can be seen from the SEM in Figure 4, the surface corrosion products in the overheated water vapor environment are uniform and dense, with aggregated large particles, and the massive protrusions are numerous and dense. Under certain conditions, the layer may fall off. EDS analysis indicated that the main elements of the inner surface were Mg, Al, Si, Ca, Cr, Mn, and Fe. From the surface scan distribution, Mg, Si, Cr, Mn, and Fe were evenly distributed and it showed aggregation of Ca and Al, which may be the cause of Ca, Mg, and Al ions in superheated water vapor. According to the energy spectrum surface analysis of Mg1.1% (mass percent, the same below), Ca 3.9%, and Al0.9%, that the water vapor quality of the boiler needs to be improved.

**Figure 5:**
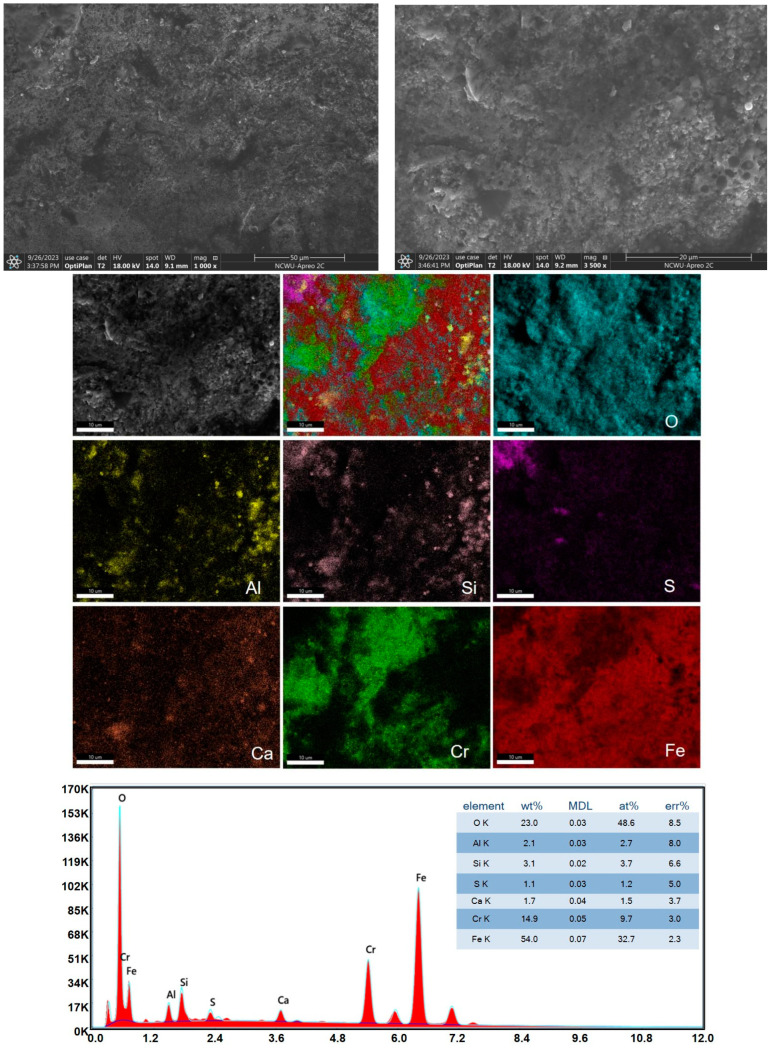
SEM pictures and EDS of the outer windward surface.

Figure 5 shows a SEM picture and EDS surface scan of the outer surface of the Super304H steel pipe after 47,000 h of service. As seen in Figure 5, the product layer is cracked and peeling, and obvious honeycomb corrosion holes occur at the bottom. The EDS analysis indicated that the main elements of the inner surface were O, Al, Si, S, Ca, Cr, and Fe. From the surface scan distribution, the atomic percent of O is close to 50% and is evenly distributed. The oxygen content of the surface aggregation sphere is higher, indicating that the sphere gathers oxide particles on the surface in the high temperature combustion chamber, and those with more Al and Si content are also those with more O content. This indicates that this is a site of alumina and silica. S exists because coal contains this element, and the corrosion of S will cause the surface to form a sheet of aggregation, which will harden the surface, consistent with the gold phase microscopy results.

**Figure 6:**
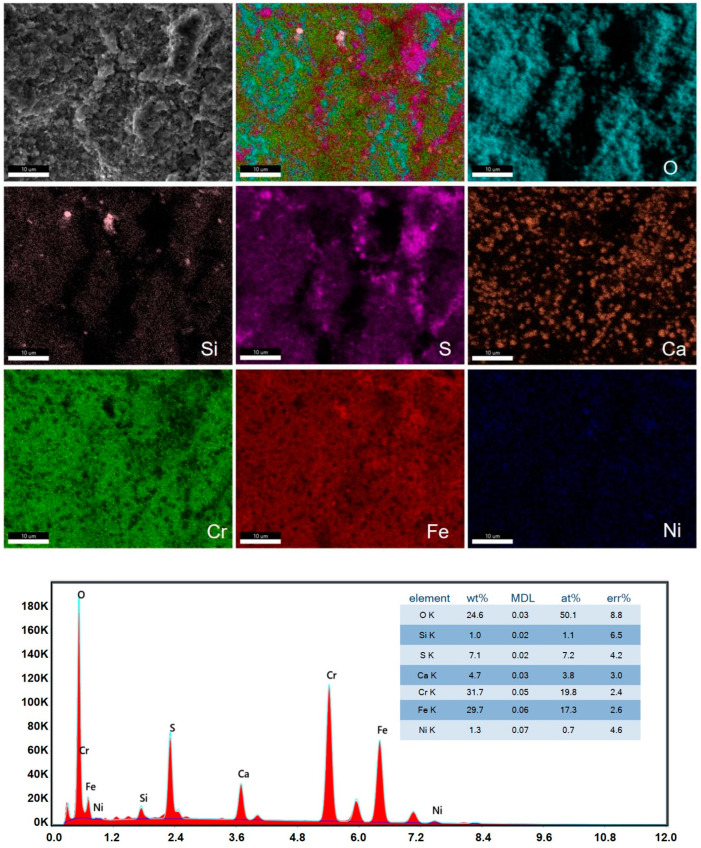
SEM pictures and EDS of the outer lee surface.

As a comparison, we measured the scanning electron microscope images and EDS surface scans on the outer surface of the Super304H steel tube after 47,000 h of service (see Figure 6). As can be seen from Figure 6, there are cracks between the products with peeling, clustered large particles, and many dense tumor processes. EDS analysis indicated that the main elements of the inner surface are O, Si, S, Ca, Cr, Fe, and Ni. From the surface scanning distribution, the atomic percentage of O is close to 50%, and the distribution is uniform, indicating that the ball is the oxide aggregation particles on the surface of the high temperature combustion chamber, Ca and S content are higher, Ca mainly has a dotted distribution, and S exists in the whole field of vision and is accompanied by an aggregation phenomenon. It shows that S is seriously attached on the leeward plane and plays a great role in the corrosion and embrittlement of stainless steel. Strengthening coal desulfurization is beneficial to extend the life of Super304H steel pipe.

Discussion: Because the affinity of Cr and O is better than other elements in Super304H steel, a Cr_2_O_3_ oxide film is formed in the air and coal ash flue gas environment. The internal metal from the film has a protective effect on the internal metal and reduces the oxidation rate. Under the long-time erosion of high-temperature air and coal ash flue gas, the continuous oxide film will crack and peel off under the thermal erosion. In this way, the corrosive S, O, Na, and other elements in the coal ash flue gas are in direct contact with the matrix metal, causing the formation of pore corrosion on the surface and acceleration of the corrosion of the metal layer, especially on the windward surface. It can be seen that under the thermal impact of the windward surface and coal ash impact, the surface oxide layer undergoes serious peeling, while there is leeward peeling. As can be seen in Figure 5 and Figure 6, the Cr content on the leeward surface is about two times that of the windward surface, and there is basically no hole erosion on the leeward surface. SO_2_ inward diffusion in coal ash flue gas in the metal layer forms oxide and sulfide. The sulfide inward diffusion goes along the boundary, and at the boundary, the sulfide will cause precipitation-phase-enhanced phase metal consumption. At the same time, the boundary sulfide formation produces large stress and corrosion under the affected part of the creep crack, and can even lead to grain cracking [17,18].

To verify the correctness discussed above, we tested the hardness of Super304H steel in the lee and windward surface, and the results are shown in Table 2 and Table 3. As can be seen from the hardness value of the lee wind surface in Table 2, the hardness of the material from the outside to inside 5 μm of Super304H steel increases first and then decreases, because the increase of S osmotic stress leads to the increase of the sub-inner hardness. In Table 3, the hardness of different positions on the windward side vary greatly, especially the hardness of the oxide layer, which is 1.5–2 times that of the oxide layer. This indicates that the oxide layer will greatly increase the hardness of the material, which becomes easy to peel off and fall off under the thermal impact and the mechanical impact of coal ash. This is in agreement with the results of Figure 2, whereby the oxide layer thickness is relatively high.

## 4. Conclusions

(1) After 47,000 h, the austenite grains at the center position of Super304H steel pipe are uniform, but some small particles tend to fuse into the large grains. With the increase of service time, even the middle part of the steel pipe also has the possibility of large grain size and performance decline. In the environment of the tube, the surface corrosion product of the tube is evenly dense, there are aggregated large particles, and the tumor protrusions are many and dense. Under certain conditions, the layer may fall off, so it is necessary to further purify the hot water to remove ions in the water as much as possible.
(2) There is an obvious corrosion layer on the surface of the inner wall of the lee plane pipe. From the corrosion layer, the stress is very large and forms a protective layer. There is an obvious stress shape between the protective layer and the inner layer, which forms a protective film to make the material more durable. There are large grains in the outer wall of the lee plane pipe, and they are more widely distributed. The fine grains are fused by coarse grains to form a large range of uncontrollable structure. In order to continue using them, their safety needs to be evaluated.
(3) The fire side of the outer wall of the windward pipe shows an obvious layered structure, which is full of holes but is relatively close to the internal structure and the non-fusion stripping layer, though slightly loose from the phase layer and the mechanical performance degradation layer. In this multi-layer structure, the hardness increases, the toughness decreases, the deformation ability is reduced, and the probability of pipe explosion is large. Compared with the leeward surface, the middle body structure is basically the same, but whether the thickness of the corrosion layer on the inner surface or the outer surface increases, the degree of deformation layer is greater. In case of pipe explosion accident, the highest chance of pipe explosion here should be closely observed.

## Figures and Tables

**Table 1:** Chemical composition of the Super304H tube (wt.%).

Elements	Fe	Cr	Ni	Mn	Si	Cu	Nb	N	C	S	B	Al
	Bal.	18.10	8.70	0.77	0.2	3.01	0.41	0.11	0.09	-	-	-

**Table 2:** Hardness of different positions of Super304H steel on the lee plane.

Hardness (Central Section) (HV)	Outer (5 μm Ecto-Entad) (HV)	Outer (10 μm) (HV)
263.1	238.1	280.1
280.2	274.6	272.3
268.1	288.7	274.9
277.9	284.4	277.1
262.8	293.3	276.4
276.9	273.9	277.3
271.5	275.1	273.6
	261.2	272.6
	271.2	270.4
	278.3	272.1

**Table 3:** Hardness of different positions of Super304H steel on the windward side.

Hardness (Central Section) (HV)	Bottom (No Oxide Layer) (HV)	Bottom (With Oxide Layer) (HV)	Left Level (HV)	Left Vertical (No Oxide Layer) (HV)	Left vertical (With Oxide Layer) (HV)	Right Level (HV)	Right Vertical (No Oxide Layer) (HV)	Right Vertical (With Oxide Layer) (HV)
261.1	268.7	580.3	266.5	426.1	385	351.9	343.9	710.4
253.4	334.4	553.9	369.3	475.4	503.2	334.4	312.9	570.3
278.1	348.8	531.4	271.5	407	693.5	319	271.3	632.9
351.7	314.3	415.5	267.2	312.9	601.7	302.3	260.6	588.5
254.0	302.9	320.0	251.0	342.1	404.1	328.7	293.9	534.4
	231.7	315.9	241.7	339.8	383.6	315.3	287.7	490.0
	232.5	316.1	252.4	354.1	361.7	281.5	281.2	484.5
	266.8	288.7	222.4	317.3	335.7	299.6	298.5	394.2
	248.7	308.5	251.6	341.2	324.8	291.7	266.7	331.3
	264.4	289.0	238.3	301.3	311.1	300.4	261.4	376.1

## Data Availability

The original contributions presented in the study are included in the article, further inquiries can be directed to the corresponding author.

## References

[1] Tang L., Huang X.T., Zeng X.Y., Zeng F. (2024). Development and Impact of Gas Power Generation Technology in Iron and Steel Companies under Dual Carbon Targets. Metall. Power.

[2] Zhang X.L. (2024). Modification and characterisation of nickel-based alloy materials for ultra-supercritical power generation. J. Phys. Conf. Ser..

[3] Yin L.F., Zhou H. (2024). Modal decomposition integrated model for ultra-supercritical coal-fired power plant reheater tube temperature multi-step prediction. Energy.

[4] Zhang C., Wang Z.Y. (2023). Comprehensive energy efficiency analysis of ultra-supercritical thermal power units. Appl. Therm. Eng..

[5] Wu Y.X., Azzi M., Khelfaoui F., Vernhes L., Martinu L., Sapieha J.K. (2024). Static friction measurement methodology for the assessment of performance of industrial valves at high temperatures: Case study for a nickel-based alloy coating. Tribol. Int..

[6] Wu Y., Chai F., Liu J.J., Wang J.Q., Li Y., Du C.C. (2024). Strengthening and Embrittling Mechanism of Super 304H Steel during Long-Term Aging at 650 °C. Materials.

[7] Xiao X.P., Li D.Z., Li Y.Y., Lu S.P. (2022). Microstructural evolution and stress relaxation cracking mechanism for Super304H austenitic stainless steel weld metal. J. Mater. Sci. Technol..

[8] Kottada R.S., Janaki Ram G.D. (2023). A critical understanding on the microstructure and creep failure of Super304H/T92 dissimilar multilayer welds. Weld. World.

[9] Zieliński A., Wersta R., Sroka M. (2022). The study of the evolution of the microstructure and creep properties of Super 304H austenitic stainless steel after aging for up to 50,000 h. Arch. Civ. Mech. Eng..

[10] Vodárek V., Kuboň Z., Palupčíková R., Hradečný K., Váňová P. (2024). Creep behaviour and microstructure evolution in Super 304H–P92 heterogeneous welds. Mater. High Temp..

[11] Kumar R., Gokhale A., Varma A., Kumar Y.R., Neelakantan S., Jain J. (2023). Role of Nb (C, N) and Cr carbides on the fracture behaviour of Super304H steel using in-situ tensile studies. Mater. Lett..

[12] Feng H.W., Mei Y.P., Tian Z. (2021). Analysis of High Temperature Reheater Tube Burst of Super304H Steel Boiler in a Power Plant. Hubei Electr. Power.

[13] Goodman S.R., Brenner S.S., Low J.R. (1973). An FIM-atom probe study of the precipitation of copper from lron-1.4 at. pct copper. Part I: Field-ion microscopy. Metall. Trans..

[14] Pizzini S., Roberts K.J., Phythian W.J. (1990). A fluorescence EXAFS study of the structure of copper-rich precipitates in Fe-Cu and Fe-Cu-Ni alloys. Phil. Mag. Lett..

[15] Osamura K., Okuda H., Ochiai S., Takashima M., Asano K., Furusaka M., Kurosawa F. (1994). Precipitation hardening in Fe-Cu binary and quaternary alloys. ISIJ Int..

[16] Wang X., Li Y., Chen D., Sun J. (2019). Precipitate evolution during the aging of Super304H steel and its influence on impact toughness. Mater. Sci. Eng. A.

[17] Leo J.R.O., Pirfo Barroso S., Fitzpatrick M.E., Wang M., Zhou Z. (2019). Microstructure, tensile and creep properties of an austenitic ODS 316L steel. Mater. Sci. Eng. A.

[18] Zhu W., Zhang Z.Y., Long D.J., Li H.J., Yu L.M. (2023). Creep deformation behavior, microstructure evolution, and damage mechanism of super304H ODS steel. Metals.

